# Microbial composition and function are nested and shaped by food web topologies

**DOI:** 10.1093/ismeco/ycaf175

**Published:** 2025-10-02

**Authors:** Samira Fatemi, Nicola G Kriefall, Danyel Yogi, Danya Weber, Nicole A Hynson, Matthew C I Medeiros, Peter Sadowski, Anthony S Amend

**Affiliations:** Pacific Biosciences Research Center, University of Hawai’i at Mānoa, Honolulu, HI 96822, United States; Pacific Biosciences Research Center, University of Hawai’i at Mānoa, Honolulu, HI 96822, United States; Pacific Biosciences Research Center, University of Hawai’i at Mānoa, Honolulu, HI 96822, United States; Pacific Biosciences Research Center, University of Hawai’i at Mānoa, Honolulu, HI 96822, United States; Pacific Biosciences Research Center, University of Hawai’i at Mānoa, Honolulu, HI 96822, United States; Pacific Biosciences Research Center, University of Hawai’i at Mānoa, Honolulu, HI 96822, United States; Information and Computer Sciences, University of Hawai’i at Mānoa, Honolulu, HI 96822, United States; Pacific Biosciences Research Center, University of Hawai’i at Mānoa, Honolulu, HI 96822, United States

**Keywords:** food webs, mosquito symbionts, microbiomes, nestedness, bromeliads, shotgun metagenomics

## Abstract

Food webs govern interactions among organisms and drive energy fluxes within ecosystems. With an increasing appreciation for the role of symbiotic microbes in host metabolism and development, it is imperative to understand the extent to which microbes conform to, and potentially influence, canonical food web efficiencies and structures. Here, we investigate whether bacteria and their taxa and functional genes are compositionally nested within a simple model food web hierarchy, and the extent to which this is predicted by the trophic position of the host. Using shotgun and amplicon sequencing of discrete food web compartments within replicate tank bromeliads, we find that both taxonomy and function are compositionally nested and largely mirror the pyramid-shaped distribution of food webs. Further, nearly the entirety of bacterial taxa and functional genes associated with hosts are contained within host-independent environmental samples. Community composition of bacterial taxa did not significantly correlate with that of functional genes, indicating a high likelihood of functional redundancy. Whereas bacterial taxa were shaped by both location and trophic position of their host, functional genes were not spatially structured. Our work illustrates the advantages of applying food web ecology to predict patterns of overlapping microbiome composition among unrelated hosts and distinct habitats. Because bacterial symbionts are critical components of host metabolic potential, this result raises important questions about whether bacterial consortia are shaped by the same energetic constraints as hosts, and whether they play an active role in food web efficiency.

## Introduction

In food webs, the transfer of energy from lower tiers like detritivores to higher tiered predators is governed by the physical laws of thermodynamics and trends toward entropy [1]. On land, this inefficiency, where approximately 90% of available energy is lost between any two adjacent levels, results in the characteristic pyramid-shaped distribution of food chain members. This fundamental constraint is well-conserved across bioregions and terrestrial habitats, ranking it among the most steadfast, longstanding, and robust rules of ecology. We now know that microbes govern the rates and efficiency with which energy is processed across environments [2, 3] and within organisms [4], so it is likely that food web efficiency and microbial function are inherently linked. Symbiotic (defined here simply as co-occurring with a host) microbial diversity and distribution are constrained by the diversity and distribution of hosts, which are, in turn, constrained by food web dynamics. Therefore, the topology of food webs should influence and be mirrored in microbial composition.

In a handful of landscape-scale studies of microbial consortia, there is some evidence linking host placement within a food web and the richness and composition of that host’s associated microbes. For example, in a study among replicated food webs in terrestrial, marine, and freshwater habitats, bacterial richness was highest at the base of the food web and declined approximately log-fold at every trophic position [5]. In other words, across habitats and along steep environmental gradients, bacterial richness serves as a sort of food web tracer, reliably indicating the trophic position of its host. Although the precise mechanism driving this pattern is undetermined, a potentially important clue is embedded in the nested composition of the microbial communities.

Nestedness—the extent to which species-poor communities represent subsets of those containing a greater number of species—is commonly observed in systems with patchy spatial structures or in landscapes where gradients of host and habitat specificity result in strong differentials of species richness [6–9]. A nested microbial topology runs counter to long held dogmas about symbiotic microbial consortia that are specific to, or co-evolved with individual hosts or substrates. If food web efficiency and complexity are constrained by the functional potential of symbiotic microbes, this might be a result of diminishing functional capacity of nested microbial communities.

The relationship between the number of species in a community and that community’s functional potential is well documented in both macro-organismal and microbial systems [10, 11]. Underpinning this phenomenon is the idea that nearly every species contains putative functional novelty. However, because the number of microbes in a given food web supersedes, by at least an order of magnitude, the number of macroorganisms, the possibility exists that microbes are functionally redundant after some critical richness threshold is achieved [12]. If some genes, traits, or functions are common among enough taxa, differences in species compositions among microbial communities may not significantly impact a function or processes of interest [12, 13].

This possibility is important for determining mechanisms underlying microbial contributions to food chain efficiency and the extent to which microbes might alter host metabolic potential. For example, in a system that is functionally redundant, log-fold differentials of microbial species richness might have little to no impact on host metabolic efficiency. Alternatively, if microbial functional potential is compositionally nested, microbial composition might play an important and unrealized role in the conversion of energy to biomass up the trophic hierarchy.

Within replicated natural, simple food webs, we tested how host trophic position shapes the composition and functional capacity of associated bacteria. We set out to test the hypothesis that bacteria are compositionally nested according to their host or habitat’s position in the food web hierarchy. We hypothesized that free-living environmental bacteria communities are the most species-rich, followed by primary producers, herbivores or omnivores, and finally carnivores. Further, we hypothesized that bacterial diversity is compositionally nested, such that the bacteria species of carnivores are a subset of those found in non-carnivorous consumers (e.g. omnivores or herbivores), which in turn are a subset of those associated with detritus, all of which are nested within the bacteria communities of other environmental substrates that serve as reservoirs for bacterial communities. We include water in our sampling because although it is not a member of the food web per se, it may serve as an important reservoir of bacteria diversity associated with mosquito hosts in early life stages [14].

While it has been argued that microbes are a critical component of any and all food webs [15], mosquito-harboring phytotelmata (aquatic plant cavity ecosystems) are one example in which the criticality of bacteria is well-documented. Mosquito larvae tend to acquire most bacteria from their local environment during their aquatic early life stages [16], and these microorganisms influence mosquito development and survivorship [17]. While bacteria or other microorganisms are not strictly required for development in all cases [18], axenic mosquito larvae display arrested or delayed development compared to those reared with microbial symbionts [17–19]. A comparison between sterile and non-sterile *Aedes aegyptii* larvae found major differences in expression of metabolic genes, which may indicate that interactions with bacteria stimulates nutrient acquisition processes [20]. Bacteria are also linked to disease resistance in bromeliad arthropods [21], and likely promote bio-availability of carbon bound in recalcitrant polymers comprising leaf litter.

To test the relationship between food web structure, bacteria community taxonomy, and bacteria community function, we generated shotgun metagenomic profiles and amplicon data among bacteria communities associated with separate trophic levels within bromeliads. We predicted that functional diversity would mirror that of taxonomic diversity and result in a nested composition that mirrors host trophic status.

## Materials and methods

### The bromeliad experimental system

**Figure 1:**
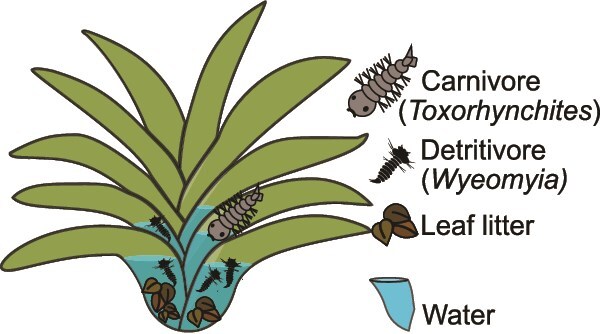
The bromeliad model system. Bromeliad contents were circumscribed and sampled as four compartments, representing distinct trophic levels. An environmental matrix: water; a primary carbon source: leaf litter; primary consumers: detritivores (predominantly *Wyeomyia* larvae); and the carnivorous *Toxorhynchites*.

To build a mechanistic understanding of how food webs impact bacterial composition and functional potential, we leveraged a bromeliad food web system (Fig. 1). For decades, bromeliads have served as natural mesocosms, providing a framework for studying temporal, spatial, and environmental impacts on biodiversity and ecosystem function [22, 23]. Bromeliad-associated invertebrates, detrital and aquatic environments, and microbes provide a tractable system for understanding microbial community assembly in the context of food webs. Tank bromeliads (*Neoregelia* sp.) at the Lyon Arboretum (21.3369444°, −157.8058333°; Honolulu, Hawaiʻi, USA) host food chains with up to three levels including leaf detritus (which provides the bulk of carbon and nutrient inputs), primary consumers (oligochaete worms and three dipteran larvae species: *Aedes albopictus*, *Culex quinquefasciatus*, and *Wyeomyia mitchellii*), and a carnivore (*Toxorhynchites amboinensis* mosquito larvae), all of which are connected within a rainwater environmental matrix. Compared to other, more diverse, bromeliad communities found in their native range, bromeliads in Hawaiʻi are simple, and the colonizing species are consistent over space and season.

### Sample collection

To sample replicate food webs, the entire contents of outdoor bromeliad phytotelmata were collected from five individual plants, located within 10 m of each other, over the course of two days using a sterile nylon baster and forceps. Samples were sorted into four compartments representing distinct trophic levels and the matrix of the food web: (i) filtered water containing free-living microbes, (ii) leaf detritus, (iii) detritivorous mosquitoes (almost exclusively *W. mitchellii*), and (iv) the carnivorous mosquito *T. amboinensis* (which preys on *W. mitchellii* larvae). Water was prefiltered with a coffee filter to exclude larvae and plant material, and filtrate was serially filtered using 5 μm, 2 μm, and 0.2 μm inline polyethersulfone filters (Sterlitech; Auburn, WA, USA) on a peristaltic pump. Detritus was homogenized in a bead beater, dried in a dehydrator, and subsampled to 0.2 g for subsequent processing. Twenty detritivorous mosquitoes were subsampled from each plant and pooled for subsequent analysis. Although we did not positively identify every detritivorous larva, *Wyeomyia* is the dominant genus at this site, and made up ~90% of the detritivore biomass in pilot studies. A single *T. amboinensis* carnivorous mosquito was collected from each phytotelma (no plant sampled contained more than a single individual). Samples were stored for up to one week at 4°C while processing, and then cryopreserved at −80°C. Sterile water was used as a negative control to identify contaminants introduced in subsequent sample processing.

### DNA extraction and sequencing

DNA was extracted using the MagAttract PowerSoil Pro DNA Kit (QIAGEN Sciences; Germantown, MD, USA) according to manufacturer’s specifications, but with the addition of a 16 h, 56°C incubation in 10 μl of Proteinase K (BioVision, cat#: AB286007-5) at 20 mg/ml concentration prior to the homogenization step. Amplicon (16S rRNA gene) library preparation was performed at the Microbial Genomics and Analytical Laboratory at the University of Hawaiʻi at Mānoa. Using oligos containing Illumina adapters, unique, sample-specific indices and primers 515FB and 806R [24–26] primers, the V4 region of the 16S rRNA gene was amplified under the following PCR conditions: 30 s of initial denaturation at 95°C; 30 cycles of 30 s of denaturation at 95°C, 60 s of annealing at 55°C, and 45 s of elongation at 68°C; and a final elongation step of 5 min at 68°C. PCR products were purified using ExoProStar (Cytiva Life Sciences; Marlborough, MA, USA) and pooled in equimolar proportions.

The resulting amplicon library was sequenced on an Illumina (San Diego, CA, USA) MiSeq platform with 2 × 300 bp paired ends (v3 chemistry) at the Advanced Studies in Genomics, Proteomics, and Bioinformatics core facility at the University of Hawai‘i at Mānoa. Shotgun metagenomics library preparation and sequencing were performed at the University of California Irvine Genomics Research and Technology Hub. Reads were sequenced as 2 × 100 bp paired ends on the Illumina (San Diego, CA, USA) NovaSeq 6000 platform to a mean sequencing depth of 10.75 GB (± 3.25 SD) per sample. Shotgun metagenomics is an imperfect measure of predicted function because the presence in a genome does not necessarily correlate with expression. Nevertheless, in some contexts gene content has been shown to significantly correlate with expressed traits (e.g. LC–MS/MS metabolite data), suggesting that it can be reasonably predictive of realized functional potential [27]. Though there are shortcomings inherent in community and functional analyses based entirely on DNA, these have the advantage of integrating comparatively long timeframes which are not indicative of momentary snapshots of activity, and enable a reasonable decoupling of functional outputs of microbes that is distinguishable from that of hosts and substrates.

### Amplicon sequencing processing

Metabarcoding of the 16S rRNA gene was used to identify bacteria present in sample types among bromeliads. All analyses were performed using R version 4.4.0 [28] and RStudio version 2024.4.2.764 [29]. Quality control of reads, inference of amplicon sequence variants (ASV), and the removal of chimeras were performed using DADA2 v.1.16 [30]. ASVs were subsequently clustered into 97% identical operational taxonomic units (OTUs) using DECIPHER [31]. Although there is no consensus about how to best to circumscribe environmental DNA sequences for community analysis, the slightly greater overlap of OTUs made analyses more straightforward here (ASVs contained <7% more taxa in this case: 1462 ASVs compared with 1370 OTUs). Taxonomy was assigned using DECIPHER compared against the SILVA SSU v138.1 database [32]. The prevalence method in Decontam v. 1.24.0 [33] was used to remove five putative contaminants. Singletons (OTUs present in only one sample across the dataset), and low-abundance count totals comprising less than 0.025% of the sample read sum were removed. These comprised ~44% of all OTUs and 0.09% of all sequence data. Finally, OTUs matching order *Chloroplast*, family *Mitochondria*, or Kingdoms other than bacteria were removed, resulting in a final dataset of 1370 OTUs.

### Shotgun metagenomics processing

Metagenomic sequences were analyzed using the SqueezeMeta pipeline v.1.6.3 [34], using the “sequential mode” of read-based annotation. Reads were trimmed with Trimmomatic v 0.4 [35] using the command “LEADING” and “TRAILING” to remove all bases below a phred quality threshold score of 30. Filtered reads were aligned using DIAMOND v.2.1.9 [36] against the Kyoto Encyclopedia of Genes and Genomes (KEGG) [37] database. Sequence homology searches were performed with HMMER v.3.4 [38] using the Pfam database [39]. We used SQMtools v.1.6.3 [40] to import data into R and to remove any reads not annotated as bacteria. A contingency table of KEGG names and annotations was constructed and standardized using the transcripts-per-million (TPM) method (which accounts for gene length). Subsequent analyses of functional diversity used the contingency table of KEGG Orthologs (KOs).

### Statistical analysis

Each of the five bromeliads sampled was considered a complete block, within which four trophic levels are experimental factors. For distance-based analyses (PERMANOVA, Mantel, and ordinations), Bray-Curtis dissimilarities were calculated from Hellinger-transformed contingency tables of the OTUs and the TPM-transformed KOs. For all other analyses, contingency tables were randomly down-sampled to contain the same sequencing depth (30 240 for 16S rRNA gene or 458 562 for KO). To estimate the extent to which sequencing effort impacted microbial diversity, we calculated accumulation curves (Supplementary Fig. S1) of observed diversity. Curves were nearly asymptotic in all samples, indicating that down-sampling will minimally impact ecological inferences in this case.

Nonmetric multidimensional scaling (NMDS) ordinations were visualized with “ggplot2” v. 3.5.1 [41]. To account for the nested experimental design, PERMANOVA analyses examined the contribution of bromeliad number (which of the five plants sampled), and the interaction between bromeliad number and trophic level (Table 1). These were calculated using the R package “vegan” v.2.6.6.1 [42].

**Table 1:** PERMANOVA results testing the effects of trophic level and bromeliad number on microbial OTU and KO variability.

**Dataset**	**Term**	**Df**	**Sum of squares**	***R*** ^**2**^	***F***	***Pr*(>*F*)**	**Significance**
OTU	Bromeliad number	1	0.65	0.10	2.68	.006	**
OTU	Trophic level × Bromeliad number	3	1.91	0.30	2.62	.001	***
OTU	Residual	15	3.64	0.58			
OTU	Total	19	6.20	1.00			
KO	Bromeliad number	1	0.03	0.02	0.69	.741	
KO	Trophic level × Bromeliad number	3	0.42	0.37	3.14	.001	***
KO	Residual	15	0.68	0.59			
KO	Total	19	1.14	1.00			

PERMANOVA results based on (A) OTU data and (B) KO abundance data. KO data are standardized using the TPM method. Both datasets were Hellinger-transformed prior to analysis. Bromeliad number indicates the plant from which the sample originates.

^***^ *P* < .001.

^**^ *P* < .01.

^*^ *P* < .05.

**Figure 2:**
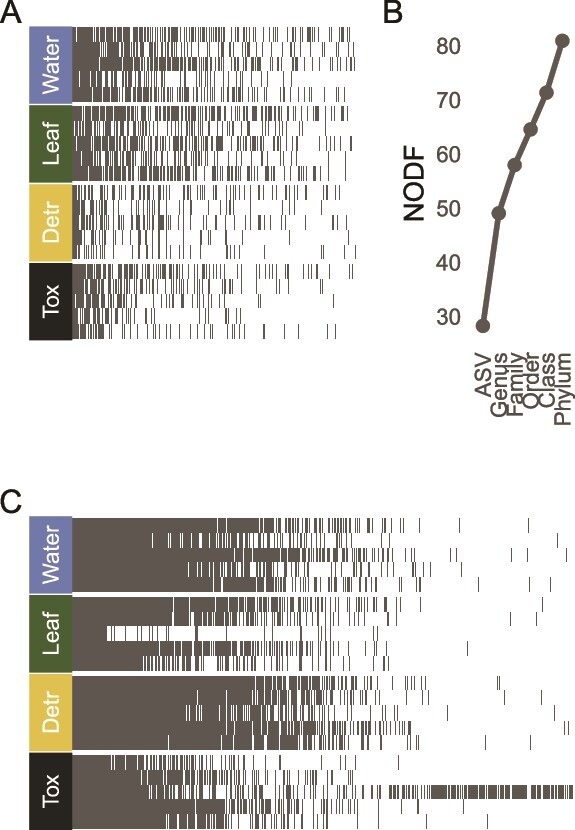
Taxa and functional attributes are nested among bromeliads. Bacterial taxa (A) and KO (B) comprised a nested hierarchy in which environmental trophic compartments contained nearly the entirety of the features found in host-associated microbiomes. Nestedness values (NODF) increased as a function of taxonomic hierarchy considered (B). Each cell indicates a feature (OTU or KO), organized by prevalence (left to right) and by richness of trophic compartment (top to bottom, richest on top).

To calculate nestedness values and their significance, we computed the nestedness based on overlap and decreasing fill (NODF) metric [43] using “nestednodf” in the “vegan” R package. Observed values were compared to a distribution of 9999 simulated null communities generated using the “r0” model of the “oecosimu” function, maintaining row sums (OTU or KO abundance distributions). Aggregate values (across the whole study) are presented in Fig. 2, with nestedness values for individual plants presented separately (Supplementary Fig. S2).

The correlation between taxonomic and functional dissimilarity of bacteria was tested using a Mantel test with Pearson correlation [44] between the pairwise dissimilarity matrices of the two contingency tables.

Linear discriminant analysis Effect Size (LefSe) analysis identified taxa and functions that best discriminate trophic levels as described in [45]. For visualization purposes, only the features among the highest 40 linear discriminant analysis (LDA) scores are presented.

## Results

### Taxonomy and function of bacteria are nested across trophic levels

**Figure 3:**
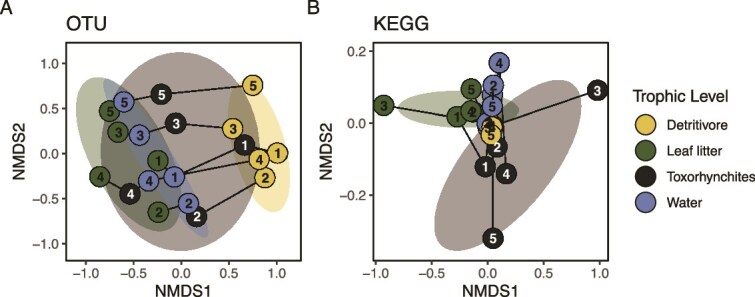
NMDS ordination plots illustrate different factors shaping microbiome taxa (A) and functions (B). Whereas both trophic compartment and bromeliad number shaped OTU composition, only trophic compartment was a significant determinant of functional gene distributions. Colors indicate trophic compartment, numbers inside circles indicate the bromeliad number.

We found that community network topologies in these four compartments in this order—water, detritus, detritivores, and *Toxorhynchites* (the carnivore)—were compositionally nested for both taxonomic and functional diversity, though to varying extents. Aggregate community taxonomic composition, at the OTU level, was nested and differed significantly from null expectations (NODF = 28.42, *P* < .001). This indicates that microbial communities with fewer species tended to comprise subsets of those found in communities with more species, consistent with a moderately nested structure. The nestedness hierarchy closely paralleled that of the food web trophic structure: most of the microbial composition of the carnivorous mosquitos comprised a subset of the diversity contained within the detritivore, which represented a subset of microbes found in the water and leaf litter (Fig. 2A). Nestedness increased as a function of taxonomic hierarchy circumscription (Fig. 2B; Phylum level NODF = 80.91). The distribution of KOs was substantially more nested (NODF = 62.497, *P* < .001; Fig. 3) than that of taxonomic composition, and generally followed the same topology, with clear divisions between the functional genes of bacteria associated with host and environmental samples.

### Taxonomic and functional diversity are shaped by different factors

Taxonomic and functional gene compositions were decoupled and predicted by different variables. Among samples, differences between functional and taxonomic community data were not significantly correlated (Mantel *r* = 0.08, *P* = .10). PERMANOVA analysis of both the taxonomic and functional compositions of samples indicated significant partitioning of variance based on food web compartment (water, detritus, detritivore, or carnivore), accounting for 75% of the explained variance in taxonomic composition and 93% of the variance in the functional composition. In contrast, bromeliad number (within the same plant, or between different plants) was only a significant determinant of taxonomic composition (Fig. 3; Table 1).

### Characteristics of the communities

**Figure 4:**
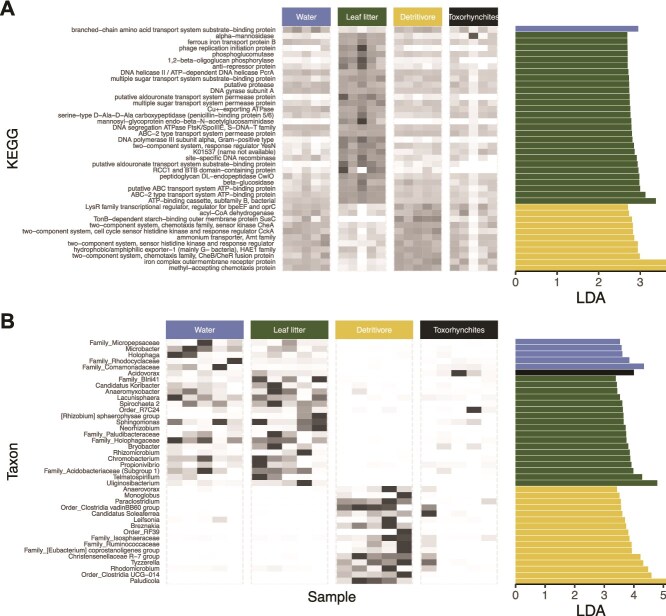
Heatmap of discriminating KO (A) and taxa (B) based on LDA effect size (LefSe; histogram at right) of features. The forty features with the highest LDA are shown here. For both datasets, detritus and primary consumers were the most discriminated, and there appears to be a division between host and environmental samples. Our analysis only considered discriminant effects on single trophic compartments, not higher-order groupings. Histograms are colored by the trophic compartment with which they are associated. Cells are shaded by proportional abundance. All taxonomic levels of the hierarchy are considered and ranked resulting in annotations of LDA that range from genus to order level circumscription.

The LDA scores were generally low, another indication of the large extent to which bacteria, and their associated functions were shared across compartments. The greatest number of differentiating taxa and genes belonged to either the detritus or detritivore trophic levels, with substantially fewer pertaining to water and carnivore compartments (Fig. 4). Detritus microbial genes were conspicuously enriched in transporter genes, whereas bacteria associated with detritivores contained a large number of genes related to chemotaxis and sensor/response.

The LDA scores for bacterial taxa were somewhat larger, although there was a greater overlap between detritus and water samples compared to the other compartments. Microbial diversity unique to detritivores generally consist of taxa associated with intestinal tracts (e.g. *Ruminocaccaceaeae*, *Clostridia*, *Paludicola*, *Tyzzerella*, *Paraclostridium*, *Monoglobus*). *Breznakia*, though not reported from mosquitoes per se, is a noted resident of termite and cockroach guts [46]. Detritus-associated bacteria include taxa that are (facultatively) anaerobic and are typical in wetland soils (e.g. *Uliginosibacterium*, *Telmatospirillum*, *Propionivibrio*, *Anaeromyxobacter* [47, 48]), or those that tend to be associated with plants and or plant decomposition (*Bryobacter*, Candidatus Koribacter, *Rhizomicrobium*, *Acidobacteriaceae*, *Neorhizobium*, *Rhizobium*, *Sphingomonas* [49, 50]).

## Discussion

As predicted, both composition and function of bacteria were nested and correlated with the trophic position of their host or environment. This result has important implications for food webs specifically, and for understanding how environments and lower trophic tiers provide reservoirs for animal hosts in a more general sense.

One implication is that microbes, when considered in a food web context, largely persist within host-independent reservoirs. This work offers a novel perspective on the dynamics of microbial community assembly. While prior studies have emphasized how microbial autecology, the environment, or host filtering shape microbiomes within related hosts or comparable environments [51–53], applying a nested food web framework highlights the potential for microbial recruitment across ecological and phylogenetic boundaries. This approach broadens the predictive scope for understanding how symbionts disperse and establish in hosts from divergent lineages or contrasting ecosystems. Bacterial functional diversity, as determined by KOs, follow a similar nested pattern (genes present in hosts are subsets of those found in non-hosts), although the slope of the relationship between trophic level and richness is shallower.

While bacterial taxonomy and function are both nested, their decoupled compositions point to functional redundancy, a second important implication. Functional redundancy is defined as “the ability of taxonomically distinct organisms to encode the same dissimilatory metabolic capabilities” [13]. Our interpretation of metagenomic data is based on the assumption that shared gene content equals shared metabolic capacity to some extent. Mantel tests indicate that taxonomic dissimilarity in bromeliads is not predictive of putative function, and unlike taxonomy, location (indicated here by bromeliad) did not explain any of the functional variance. In other words, although different bacteria occurred in a given trophic level among replicate bromeliads, those bacteria tended to contain a similar combination of functional genes.

Previous work among bromeliad detrital samples found similar divergences between taxonomic and functional distributions, with geography explaining only a negligible amount of variance in a minority of bacteria functional groups [54]. This pattern of conserved function and divergent taxonomy appears to be common in symbiotic microbial consortia in systems as divergent as human guts to pitcher plants to mycorrhizal fungi [55–57], and is one way by which microbial symbioses persist despite dispersal limitation. Bromeliad food webs are doubly redundant, because functional potential is consistent among bromeliads, but also within them, as the majority of microbial function genes associated with symbioses are also present in environmental samples.

Bio-accumulation and metacommunity dynamics are two different, though non-exclusive mechanisms that might contribute to the nestedness topology. Bio-accumulation, the first mechanism, can be referred to as the “you are what you eat” model, in which microbes are passed up the food chain inefficiently (via consumption or other modes of transmission). A diminishing minority of microbes survive each serial passage, resulting in ordered extinctions that form a nested topology.

Metacommunity dynamics is our second proposed mechanism for understanding the nested composition of microbial communities across trophic levels within bromeliad food webs. In our study system, bromeliad water functions as a shared, hospitable matrix that physically connects multiple habitat patches—namely, trophic compartments such as detrital pools, detritivores, and predators—within a single metacommunity [58]. This aqueous matrix facilitates microbial dispersal among compartments, while simultaneously allowing for distinct selective pressures within each patch. The basal detrital pool presents a highly heterogeneous and nutrient-rich environment, supporting a diverse and functionally broad microbial assemblage. As microbial taxa move upward through the food web—from detritus to detritivores and ultimately to predators—communities may become progressively filtered, resulting in nested subsets shaped by increasingly restrictive niche conditions, altered physicochemical environments, and host-derived selection [59]. Thus, while dispersal is largely unimpeded by physical barriers due to the connectivity of bromeliad water, species-sorting dynamics likely dominate the structuring process, producing a predictable pattern of nestedness. This perspective aligns with modified metacommunity models that explicitly incorporate microbiome biology, in which within-host filtering and trophic interactions lead to non-random, hierarchical assembly patterns across spatially and biologically linked habitats [59, 60].

It bears mentioning that while a nested topology reflects a pyramid-shaped sampling scheme, the results are not merely an artifact of it. The laws of thermodynamics ensure that there is no ecosystem on Earth in which biomass is equivalent among trophic levels. Nevertheless, in our study, total sampled biomass was consistent among sampled phytothelmata. Further, sampling saturation models across trophic levels using extrapolated collectors’ curves (Supplementary Fig. S1) demonstrate that there was no systematic undersampling (via sequencing effort) that might skew observed richness patterns.

Nestedness of microbial taxa is a common occurrence in microbial community ecology [5, 16, 61–63], particularly at landscape scales. Using a simple, replicated model food web, we show that bacteria within bromeliads are nested and their hierarchical order is correlated with the trophic level of the sampled host. Although gene content was similarly nested, there was no significant correlation between bacterial taxonomy and functional composition, and variables predicting these assemblages were distinct from each other. While bacterial taxa were constrained by location, functional potential was not. Parameterizing microbiome composition and function within the theory of food web dynamics enables a rational framework to predict microbial assembly and distribution among seemingly disconnected hosts and habitats. Metabolic efficiencies lead to pyramid-shaped food webs, which are similar to decreasing patterns of microbial richness and functional potential. Whether microbial composition contributes to this hierarchy, or is instead shaped by it while responding to the same laws of thermodynamics as hosts, is an exciting and consequential area for further experimental investigation.

## Supplementary Material

FigS1_ycaf175

FigS2_ycaf175

Metadata_csv_ycaf175

## Data Availability

The 16S rRNA gene and shotgun DNA sequences generated in this study can be found on the NCBI database at BioProject ID number PRJNA1181938. A metadata table containing characteristics of bromeliads and samples is appended here in the supplements. All coding scripts and intermediate processing objects are available at (github.com/anthonyamend/Bromeliad5.git).
