# Supplementary material for: Microbial composition and function are nested and shaped by food web topologies
Source: ISME Commun. 2025 Oct 2;5(1):ycaf175. doi: 10.1093/ismeco/ycaf175 (PMC12558044; doi:10.1093/ismeco/ycaf175)
Supplement: FigS1_ycaf175 [file figs1_ycaf175.pdf]

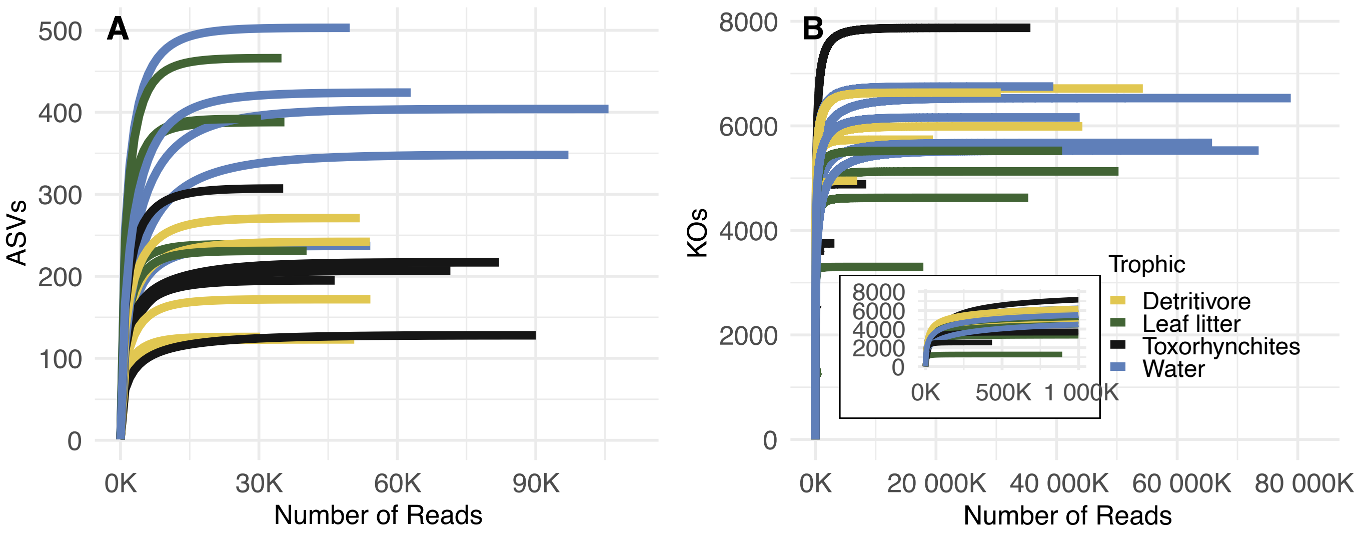

Figure S1. Rarefaction curves of OTUs (A) and KOs (B) approach asymptotes, suggesting that sequencing depth was adequate to nearly census diversity within samples. Inset figure in panel B is the same data, but with a magnified X axis to demonstrate saturation of samples with lower sequencing depth.
