# Supplementary material for: Microbial composition and function are nested and shaped by food web topologies
Source: ISME Commun. 2025 Oct 2;5(1):ycaf175. doi: 10.1093/ismeco/ycaf175 (PMC12558044; doi:10.1093/ismeco/ycaf175)
Supplement: FigS2_ycaf175 [file figs2_ycaf175.pdf]

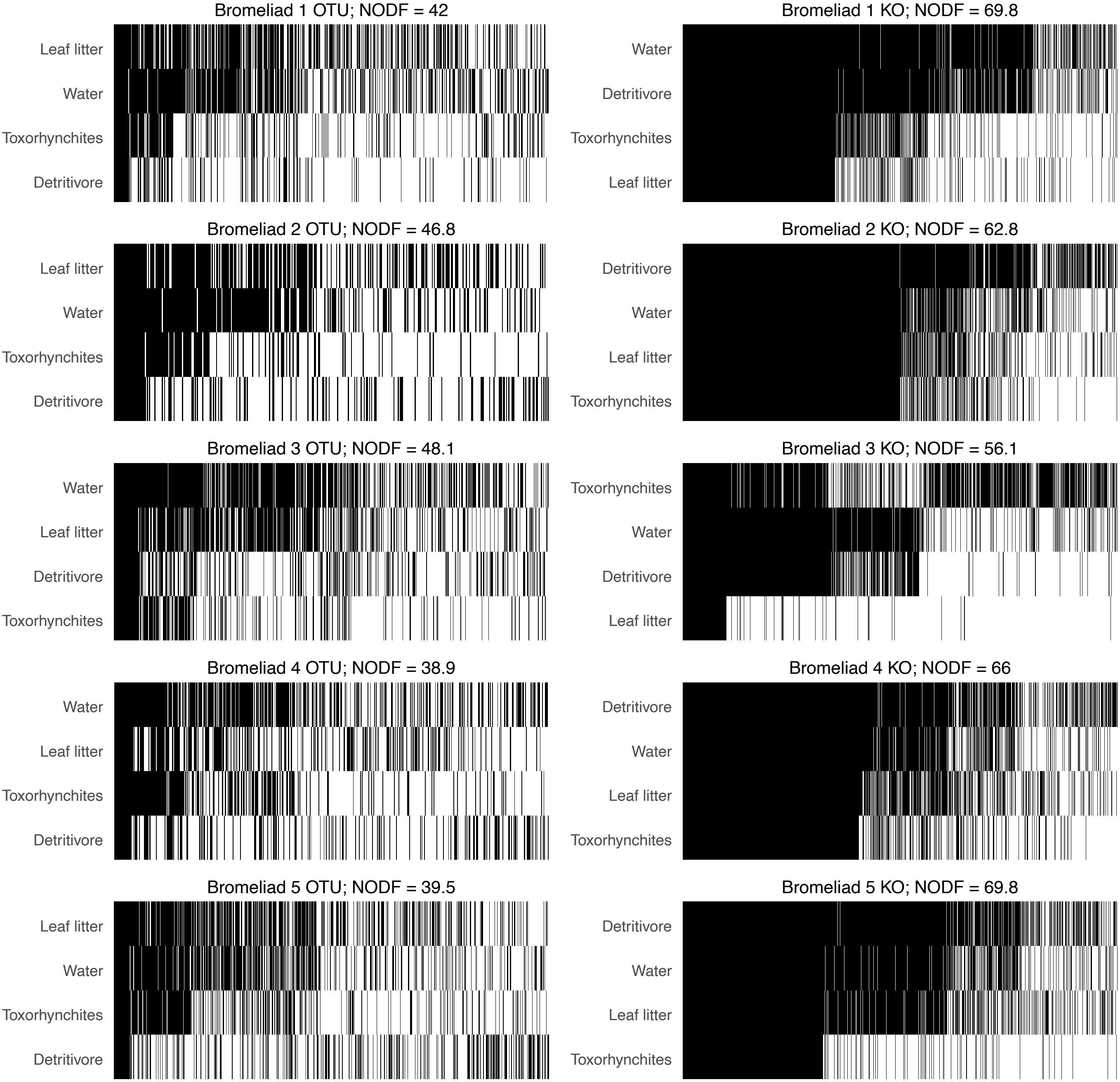

Figure S2. Nestedness plots by bromeliad. Tile plot indicates the presence or absence of features, organized by row sums (top to bottom) and column sums (left two right). The same plant occupies both columns of the same row, with OTU composition on the left, and KO data on the right. NODF values are reported on the binary data.
